# Self-reported diabetes or hypertension diagnoses and antenatal care among child-bearing women in rural Bangladesh: A cross-sectional study

**DOI:** 10.1371/journal.pgph.0002175

**Published:** 2023-09-14

**Authors:** Allyson P. Bear, Wendy L. Bennett, Joanne Katz, Kyu Han Lee, Atique Iqbal Chowdhury, Sanwarul Bari, Shams El Arifeen, Emily S. Gurley

**Affiliations:** 1 Department of International Health, Johns Hopkins Bloomberg School of Public Health, Baltimore, Maryland, United States of America; 2 Department of Medicine, Division of General Internal Medicine, Johns Hopkins School of Medicine, Baltimore, Maryland, United States of America; 3 Division of Maternal and Child Health, International Centre for Diarrheal Disease Research, Bangladesh, Dhaka, Bangladesh; American University of Beirut, LEBANON

## Abstract

Health care systems in low- and middle-income countries may not meet the needs of pregnant women where the burden of diabetes and hypertension is rapidly increasing. We asked recently pregnant women about ever having been screened for or diagnosed with hypertension or diabetes and their ANC-seeking experiences in a cross-sectional survey. We used chi-squared tests and logistic regression to test the associations between self-reported coverage of hypertension and diabetes screening, diagnoses, and elements of ANC by age, wealth, educational attainment, and gravidity. Among 4,692 respondents, for hypertension, 97% reported having been screened and 10% of screened women reported a diagnosis. Women 30–39 years of age (aOR 3.02, 95% CI 2.00, 4.56) or in the top wealth quintile (aOR 1.70, 95% CI 1.18, 2.44) were more likely to be diagnosed with hypertension compared to reference groups. Any hypertension diagnosis was associated with reporting four or more antenatal care contacts (44% vs. 35%, p < 0.01), blood pressure measurements (85% vs. 79%, p < 0.01), and urine tests (71% vs. 61%, p < 0.01) conducted during ANC visits. For diabetes, 46% of respondents reported having been screened and 3% of screened women reported a diagnosis. Women 30–39 years of age were more likely to be diagnosed with diabetes (aOR 8.19, 95% CI 1.74, 38.48) compared to the reference group. Any diabetes diagnosis was associated with reporting four or more ANC contacts (48% vs. 36%, p = 0.04) and having blood testing during pregnancy (83% vs. 66%, p < 0.01). However, the frequency and quality of ANC was below the national guidelines among all groups. Focused efforts to ensure that women receive the recommended number of ANC contacts, coupled with improved compliance with ANC guidelines, would improve awareness of hypertension and diabetes among women in Bangladesh.

## Introduction

The prevalence of diabetes and hypertension is rising in low- and middle-income countries. Globally, the number of people living with diabetes has risen from 151 million in 2000 to 463 million in 2019; of these, 79% live in low- or middle-income countries [1]. The number of people living with hypertension has risen from 932 million in 2000 to 1.4 billion in 2010 [2,3]. Growing urbanization, changing lifestyle habits, and genetic factors are some of the reasons for this increase in low- and middle-income countries [1–3]. South Asia accounts for 60% of the global diabetes and 23% of the global hypertension burdens, and these health conditions play increasing roles in pregnancy-related morbidity and mortality [4–9]. Hyperglycemia, including pre-existing and gestational diabetes, is estimated to complicate 17% of all pregnancies globally; 9 out of 10 of these cases occur in less developed countries [5]. In underdeveloped health care systems, the risk of perinatal mortality is 2.5–5 times higher for women with pre-existing diabetes as compared to women without, and an estimated 50% of neonates born to women with the condition require admission to intensive care units [10–13]. Hypertensive disorders of pregnancy are estimated to complicate 5–10% of all pregnancies globally and are responsible for an estimated 16% of stillbirths and 10% of all early neonatal deaths [9,14–16]. These two conditions can also create detrimental synergies; for example, mothers with pre-existing diabetes are also at a higher risk of hypertensive disorders during pregnancy, including a nine-times greater risk of developing pre-eclampsia [6,11,17,18].

Diabetes and hypertension are the major causes of morbidity and mortality in Bangladesh, including maternal mortality, 24% of which is attributable to pre-eclampsia or eclampsia [19–21]. From 2011 to 2018, hypertension prevalence increased from 32% to 45% among women over 35 years of age and was estimated to be 12.5% among women 18–34 years of age in 2018 [22]. The burden of pregnancy-induced or primary hypertension in pregnancy is less well understood, as is its impact on pregnancy outcomes other than maternal mortality. Similarly, the prevalence of diabetes is also increasing in Bangladesh; from 2011 to 2018, it increased from 12% to 14% among women over 35 years of age and was estimated to be 5% among women 18–34 years of age [22]. An estimated 13% of women in rural Bangladesh develop gestational diabetes mellitus during pregnancy [23]. Since 2016, national guidelines for maternity care in Bangladesh have included screening for both hypertension and diabetes as part of routine antenatal care, but the extent to which these services are provided to women in pregnancy is not well-documented [24]. The objective of this study was to describe the self-reported prevalence of screening and diagnoses of diabetes and hypertension among recently pregnant women in a rural area of Bangladesh and the antenatal care received by women with self-reported diabetes and hypertension during their pregnancies.

## Methods

### Ethics statement

All methods were carried out in accordance with relevant guidelines and regulations. Ethical approval was provided by the Ethical Review Committee of the International Centre for Diarrheal Disease Research, Bangladesh on April 24, 2019 under protocol number PR-19023. Additional information regarding the ethical, cultural, and scientific considerations specific to inclusivity in global research is included in the Supporting Information (S1 Checklist).

### Data and methods

This study was conducted at the Child Health and Mortality Prevention Surveillance (CHAMPS) project site in the Baliakandi sub-district of Bangladesh. It is a rural, agrarian area of the country. Within the sub-district there are 7 public sector health clinics and 1 sub-district hospital. Several public and private secondary and tertiary hospitals are located in two district capital towns between 1–1.5 hours travel time by vehicle. CHAMPS Bangladesh began active population-based demographic surveillance in the sub-district in September 2017. In 2017 Baliakandi sub-district had 261 villages and a total population of 220,000, including approximately 56,000 women of reproductive age and 19,500 children under five years of age [25,26]. CHAMPS uses information from four primary sources of data to monitor population health and child mortality: health care facility-based death notification, community-based death notification, pregnancy surveillance, and demographic surveillance. All households within in Baliakandi sub-district are included in the surveillance activities.

Within the context of the CHAMPS active population-based surveillance, from April to August 2019, we conducted a cross-sectional survey of married women of reproductive age to ascertain prior screening for and diagnosis of hypertension and diabetes. Within the CHAMPS catchment area under active surveillance, all households with a child (living or dead) under five years of age or a woman who was pregnant were eligible to participate. One week prior to the start of data collection in each block of the CHAMPS demographic surveillance system, a listing of households that met the eligibility criteria for the cross-sectional survey was generated using the CHAMPS data on pregnancies and children under five years of age. Data collectors visited each household and conducted face-to-face interviews. Written informed consent was obtained from the woman, or, in the event that the woman was under the age of 18 years, informed assent was taken and witnessed by a guardian from the household. If a woman eligible for participation was not at home during the initial visit, data collectors conducted up to nine follow-up visits to complete the data collection for the cross-sectional survey.

The cross-sectional survey contained two modules used in this study: maternal hypertension and diabetes, and antenatal care. The data collection tool was based on questionnaires developed by the Demographic and Health Survey (DHS) Program and the WHO STEPwise approach to surveillance [27,28]. The questions were translated into Bengali and validated through prior national surveys [29–31]. Using structured questions, the data collectors asked eligible women about previous screening and diagnoses of diabetes and hypertension, the timings of diagnoses, and having received each of the following antenatal services at least once at any point in their pregnancy: height, weight, and blood pressure measurements; urine tests (unspecified); blood tests (unspecified); calcium supplements; iron supplements; and tetanus toxoid vaccinations. No medical records were available to confirm the self-reported information.

### Data analysis

Using the demographic surveillance information available as of February 26, 2020, we retrospectively identified a subset of individual women with pregnancy outcomes that occurred within the 12 months prior to the date of the cross-sectional survey. Using the same data set, we extracted demographic, socio-economic, and pregnancy history information from the demographic surveillance database for each survey respondent by linking unique identification numbers. All data used for this study were deidentified.

Using summary statistics and chi-squared tests, we examined the following variables for each eligible respondent: demographic and socio-economic characteristics, including age (< 20, 20–29, 30–39, and 40+ years of age), wealth quintile, and educational attainment (none, primary, secondary, and post-secondary); health history variables, including gravidity (the total number of lifetime pregnancies), diabetes, and hypertension; and care-seeking in pregnancy, including the number of antenatal care visits (0, < 4, 4–8, and 9+) and elements of antenatal care. The wealth quintile was constructed using the DHS wealth index score [32,33]. Based on a literature review of known risk factors for hypertension, hypertensive disorders of pregnancy, diabetes, and hyperglycemia in pregnancy, we controlled for age, wealth quintile, educational attainment, and gravidity as potential confounders in the analysis [34–40]. We used logistic regression to estimate the associations between selected background characteristics and diabetes or hypertension screening and adjusted for known confounders. We then used logistic regression to estimate the associations between diabetes or hypertension diagnoses and selected background characteristics among women who had been previously screened, adjusting for the same confounders. We used chi-squared tests to examine the associations between previous diabetes or hypertension diagnoses and the measured elements of antenatal care. All variables were analyzed categorically. A value of p < 0.05 was considered statistically significant for all analyses. Data analysis was performed using Stata 14 statistical software.

## Results

**Fig 1:**
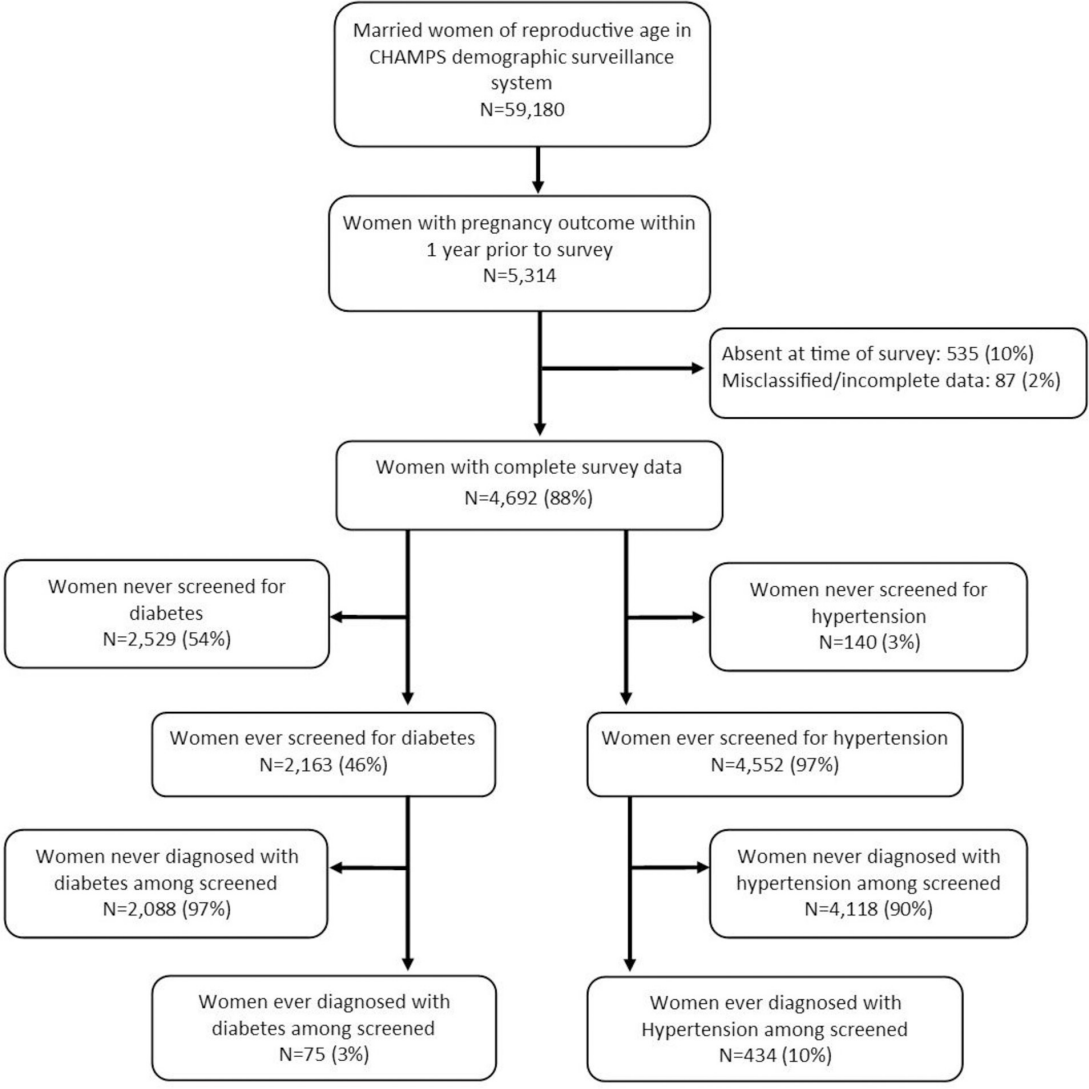
Analytic cohort of women in study, Baliakandi, Bangladesh, 2019.

Among 59,180 married women of reproductive age, we identified 5,314 women with any documented pregnancy outcome within one year prior to the survey (Fig 1). Pregnancy outcomes could include stillbirth, live birth, or miscarriage/abortion. The overall response rate was 90%. Among respondents, 87 (2%) were excluded from analysis due to missing survey information. A total of 4,692 women (88% of the eligible population) were included in the analysis (Fig 1). Approximately 46% (2,163 out of 4,692) of respondents reported previously having been screened for diabetes, compared to nearly all having been previously screened for hypertension (97%). Of those screened, 3% (75 out of 2,163) reported previous diagnoses of diabetes, and 10% (434 out of 4,552) reported previous diagnoses of hypertension (Fig 1).

Most recently pregnant women (78%) were under 30 years of age, and 35% had recently completed their first pregnancy (Table 1). We observed a prominent generational difference in educational status among the women surveyed: 72% of women over 40 years of age reported primary school completion or lower, while 89% of women under 20 years of age reported secondary school completion or higher (S1 Table).

**Table 1:** Age, socio-economic and health history characteristics of the study population, Baliakandi, Bangladesh, 2019.

Characteristic	All women n(%)
	N = 4,692
**Age (Years)**	
<20	925 (20%)
20–29	2,707 (58%)
30–39	1,021 (22%)
40–49	39 (<1%)
**Education (Completed)**	
None	202 (4%)
Primary	873 (19%)
Secondary	2,406 (51%)
Post-Secondary	1,211 (26%)
**Household Wealth Quintile**	
Lowest	909 (19%)
Second	921 (20%)
Middle	949 (20%)
Fourth	981 (21%)
Highest	932 (20%)
**Gravidity (the total number of lifetime pregnancies)**	
1	1,651 (35%)
2–4	2,765 (59%)
5+	276 (6%)
**Diabetes**	
Ever screened	4,552 (97%)
Ever diagnosed among screened	75(3%)
**Hypertension**	
Ever screened	2,163(46%)
Ever diagnosed among screened	434(9%)
**Number of Antenatal Care Contacts**	
None	541(12%)
< 4	2,460(52%)
4–8	1,537(33%)
9+	154(3%)

### Characteristics associated with diabetes and hypertension screening

In crude and adjusted analyses, young women and women with no education had the lowest odds of ever having been screened for diabetes compared to other groups (Table 2). After adjusting for age, education, wealth, and gravidity, primigravid or multigravida (5+) women below the fourth wealth quintile had significantly lower odds of ever having been screened for diabetes compared to wealthier women and women reporting a lifetime total of 2–4 pregnancies (Table 2).

**Table 2:** Association between diabetes and hypertension screening and selected background characteristics using logistic regression, Baliakandi, Bangladesh, 2019.

Characteristics		Diabetes					Hypertension			
	n(%)^	Crude		Adjusted		n(%)^	Crude		Adjusted	
	4,692	OR	95% CI	aOR	95% CI	4,692	OR	95% CI	aOR	95% CI
**Age (Years)**										
< 20	353(38)	Ref		Ref		886(96)	Ref		Ref	
20–29	1,251(46)	1.39*	(1.19, 1.62)	1.23*	(1.03, 1.47)	2,639(97)	1.71*	(1.14, 2.55)	1.49	(0.92, 2.42)
30–39	536(52)	1.79*	(1.49, 2.15)	1.80*	(1.44, 2.27)	988(97)	1.32	(0.82, 2.11)	1.33	(0.70, 2.51)
40–49	23(59)	2.33*	(1.21, 4.47)	2.93*	(1.44, 5.92)	39(100)	--	--	--	--
**Education (Completed)**										
None	64(32)	Ref		Ref		193(95)	Ref		Ref	
Primary	362(41)	1.53*	(1.10, 2.11)	1.71*	(1.23, 2.39)	839(96)	1.15	(0.54, 2.44)	1.24	(0.58, 2.64)
Secondary	1,068(44)	1.72*	(1.27, 2.34)	2.15*	(1.56, 2.97)	2,334(97)	1.51	(0.74, 3.07)	1.79	(0.85, 3.74)
Post-Secondary	669(55)	2.66*	(1.94, 3.66)	3.27*	(2.32, 4.60)	1,186(98)	2.21*	(1.02, 4.81)	2.46*	(1.06, 5.70)
**Household Wealth Quintile**										
Lowest	367(40)	Ref		Ref		882(97)	Ref		Ref	
Second	403(44)	1.15	(0.95, 1.38)	1.16	(0.96, 1.33)	891(97)	0.91	(0.54, 1.54)	0.92	(0.54, 1.56)
Middle	415(44)	1.15	(0.95, 1.38)	1.10	(0.91, 1.33)	914(96)	0.80	(0.48, 1.33)	0.74	(0.44, 1.24)
Fourth	473(48)	1.37*	(1.15, 1.65)	1.26*	(1.04, 1.52)	954(97)	1.08	(0.63, 1.86)	0.97	(0.56, 1.69)
Highest	505(54)	1.75*	(1.45, 2.10)	1.38*	(1.14, 1.69)	911(98)	1.33	(0.74, 2.37)	1.04	(0.57, 1.91)
**Gravidity**										
1	707(43)	Ref		Ref		1,594(96)	Ref		Ref	
2–4	1,323(48)	1.22*	(1.08, 1.38)	1.20*	(1.03, 1.40)	2,693(97)	1.34	(0.94, 1.90)	1.32	(0.83, 2.09)
5+	133(48)	1.24*	(0.96, 1.60)	1.16	(0.86, 1.57)	265(96)	0.86	(0.45, 1.67)	0.93	(0.41, 2.10)

Adjusted model includes age, education, wealth, and gravidity.

*Denotes significance at the p < 0.05 level.

^Percent screened out of the total number of women in the category.

All women over 40 years of age reported having previously been screened for hypertension at least once in their lives. Women who had completed post-secondary education were two-fold more likely to report having been previously screened for hypertension (OR 2.21, 95% CI 1.02, 4.81), and this association strengthened after controlling for age, wealth, and gravidity (aOR 2.46, 95% CI 1.06, 5.70) (Table 1). Among the respondents, 3% (140 of 4,692) reported having never been screened for hypertension; 41% (57 out of 140) were primigravida. Overall, having never been screened was associated with having very little interaction with the health care system during pregnancy; 46% (65 out of 140) reported either receiving no antenatal care or having an ultrasound as their only antenatal care during pregnancy.

### Diabetes and hypertension diagnoses

While higher educational attainment and increased wealth were associated with an increased likelihood of ever having been screened for diabetes (Table 3), these characteristics were not associated with higher odds of reporting a diagnosis of diabetes in adjusted analyses (Table 3). Membership in the highest wealth quintile (aOR 1.73, 95% CI 1.24, 2.43) was the only statistically significant socio-economic factor associated with increased risk for hypertension in fully adjusted analyses.

**Table 3:** Association between diabetes and hypertension diagnoses and selected background characteristics using logistic regression, Baliakandi, Bangladesh, 2019.

Characteristics	Diabetes					Hypertension				
	
	n(%)^	Crude		Adjusted		n(%)^	Crude		Adjusted	
	N = 2,163	OR	95% CI	aOR	95% CI	N = 4,552	OR	95% CI	aOR	95% CI
**Age (Years)**										
< 20	2(<1)	Ref		Ref		48(5)	Ref		Ref	
20–29	34(3)	4.90*	(1.17,20.51)	3.79	(0.84,17.02)	216(8)	1.56*	(1.13,2.15)	1.44*	(1.01,2.08)
30–39	39(7)	13.77*	(3.30,57.41)	8.19*	(1.74,38.48)	162(16)	3.42*	(2.45,4.79)	3.02*	(2.00,4.56)
40–49	0(0)	-	--	-	--	8(20)	4.50*	(1.97,10.33)	3.37*	(1.36,8.31)
**Education (Completed)**										
None	5(8)	Ref		Ref		24(12)	Ref		Ref	
Primary	16(4)	0.55	(0.19,1.55)	0.67	(0.23,1.92)	86(10)	0.80	(0.50,1.30)	0.94	(0.58,1.55)
Secondary	39(4)	0.45	(0.17,1.78)	0.69	(0.26,1.89)	207(9)	0.68	(0.44,1.08)	0.99	(0.62,1.60)
Post-Secondary	15(2)	0.27	(0.17,1.18)	0.44	(0.14,1.38)	117(10)	0.77	(0.48,1.23)	1.08	(0.65,1.82)
**Household Wealth Quintile**										
Lowest	14(4)	Ref		Ref		68(8)	Ref		Ref	
Second	14(3)	0.91	(0.43,1.93)	0.96	(0.45,2.07)	86(10)	1.28	(0.92,1.78)	1.35	(0.96,1.89)
Middle	12(3)	0.75	(0.34,1.65)	0.83	(0.37,1.83)	80(9)	1.15	(0.82,1.61)	1.22	(0.86,1.71)
Fourth	13(3)	0.71	(0.33,1.53)	0.91	(0.41,2.01)	85(9)	1.17	(0.84,1.63)	1.26	(0.89,1.77)
Highest	22(4)	1.15	(0.58,2.28)	1.51	(0.72,3.17)	13(115)	1.73*	(1.26,2.37)	1.73*	(1.24,2.43)
**Gravidity**										
1	9(1)	Ref		Ref		112(7)	Ref		Ref	
2–4	55(4)	3.36*	(1.65,6.85)	1.66	(0.74,3.69)	273(10)	1.49*	(1.19,1.88)	1.09	(0.82,1.44)
5+	11(8)	6.99*	(2.84,17.23)	2.40	(0.84,6.91)	49(18)	3.00*	(2.08,4.32)	1.56	(0.99,2.44)

Adjusted model includes age, education, wealth, and gravidity.

* denotes significance at the p<0.05 level

^Percent diagnosed out of total number of women ever screened in the category.

In the fully adjusted analyses, a higher age was significantly associated with higher odds of diagnoses of both hypertension and diabetes compared to a lower age of < 20 years. Women 30–39 years of age had significantly higher odds of hypertension (aOR 3.02, 95% CI 2.00, 4.56) and diabetes (aOR 8.19, 95% CI 1.74, 38.48) diagnoses compared to women under 20 years of age (Table 3). Among the 39 recently pregnant women over 40 years of age (Table 1), 23 (59%) had ever been screened for diabetes, and none reported a history of diabetes diagnosis. Women over 40 years of age had the highest odds of hypertension diagnosis (aOR 3.37, 95% CI 1.36, 8.31) compared to women under 20 years of age. The number of total lifetime pregnancies was not associated with higher odds of hypertension or diabetes diagnoses in the adjusted analyses (Table 3).

### Antenatal care services among women with diabetes and hypertension

Among women with reported diabetes diagnoses, 53% (40 out of 75) occurred before and 47% (35 out of 75) occurred during or after the index pregnancy (Table 4). Women with any diabetes diagnosis were more likely to have four or more antenatal care contacts compared to women who were never diagnosed (48% vs. 36%, p = 0.04). Women with any diabetes diagnosis were significantly more likely to report having blood tests during antenatal care compared to women who were never diagnosed (83% vs. 66%, p < 0.01) (Table 4). A greater proportion of women with any diabetes diagnosis reported receiving calcium and iron folate supplements, any urine test, and having their weight and blood pressure measured compared to women who have never been diagnosed, but these differences were not statistically significant (Table 4). Among women with any diabetes diagnosis, 17% (13 out of 75) received all seven measured elements of antenatal care, including 15% (8 out of 53) of women diagnosed with diabetes prior to the index pregnancy.

**Table 4:** Antenatal care services among women with diabetes and hypertension, Baliakandi, Bangladesh, 2019.

	Diabetes		P-value	Hypertension		P-value
	No n(%)	Yes n(%)		No n(%)	Yes n(%)	
	N = 4,617	N = 75		N = 4,258	N = 434	
**Number of antenatal care contacts**			0.04*			<0.01*
None	535 (12)	6 (8)		502 (12)	39 (9)	
< 4	2,427 (53)	33 (44)		2,257 (53)	203 (47)	
4–8	1,507 (33)	30 (40)		1,360 (32)	177 (41)	
9+	148 (3)	6 (8)		139 (3)	15 (3)	
**Elements of antenatal care**						
Weight Taken	3,486 (76)	63 (84)	0.09	3,206 (75)	343 (79)	0.08
Blood Pressure Taken	3,648 (79)	65 (87)	0.11	3,343 (79)	370 (85)	<0.01*
Any Urine Test	2,857 (62)	51 (68)	0.28	2,599 (61)	309 (71)	<0.01*
Any Blood Test	3,070 (66)	62 (83)	<0.01*	2,814 (66)	318 (73)	<0.01*
Tetanus Toxoid Vaccine	2,092 (45)	27 (36)	0.27	1,946 (46)	173 (40)	0.05
Any Iron Folate Supplement	3,202 (69)	55 (73)	0.75	2,944 (69)	313 (72)	0.42
Calcium Supplement	3,107 (67)	54 (72)	0.68	2,863 (67)	298 (69)	0.29
Received all seven measured elements of care	1051 (23)	13 (17)	0.27	97 (22)	97 (22)	0.86

The p-value compares women ever diagnosed to women never diagnosed with the disease.

* denotes significance at the p < 0.05 level.

Among women with reported hypertension diagnoses, 36% (158 out of 434) occurred before and 64% (276 out of 434) occurred during or after the index pregnancy (Table 4). Women with any hypertension diagnosis were more likely to have four or more antenatal care contacts compared to women who have never been diagnosed (44% vs. 35%, p < 0.01). Women with any hypertension diagnosis were significantly more likely to receive calcium supplements and tetanus toxoid vaccination, report having their weight and blood pressure measured, and have any blood test during pregnancy compared to women who have never been diagnosed (Table 4). Among women with any hypertension diagnosis, 22% (97 out of 434) received all seven measured elements of antenatal care, including 15% (28 out of 158) of women diagnosed with hypertension prior to the index pregnancy.

Among the respondents, 8% of diabetes and 19% of hypertension diagnoses occurred after delivery, indicating that the disease may not have been identified through routine antenatal care in pregnancy. Despite more antenatal contacts during pregnancy, after adjusting for age, education, wealth, and the total number of lifetime pregnancies, women with any diagnosis of diabetes (aOR 0.87, 95% CI 0.47, 1.60) or hypertension (aOR 1.05, 95% CI 0.82, 1.34) were no more likely to receive all seven measured elements of antenatal care services at least once in their pregnancy compared to never diagnosed women.

## Discussion

Nearly all the women in this study 97% reported ever having been screened for hypertension, and nearly half (46%) reported ever having been screened for diabetes. Per the national guidelines for antenatal care, all study respondents should have been screened for hypertension, with a medical history of hypertension and diabetes taken at their first antenatal care visit followed by blood pressure readings at each subsequent visit and a blood glucose test between 24 and 28 weeks of pregnancy [41]. Among women in this study, 79% had their blood pressure checked at an antenatal care visit, and 67% reported having some type of blood test as part of their antenatal care during the index pregnancy. These rates of screening are higher than in other national surveys, which could suggest that antenatal care is a primary source of hypertension and diabetes screening for women in rural Bangladesh [22,29,31]. Our findings suggest that diabetes screening may be offered selectively, based on risk factors (such as age), patient advocacy, or the choice of facility, depending on the patient’s socio-economic status. This hypothesis merits further research. Nearly 9 out of 10 women interacted with the health system at least once to receive antenatal care during pregnancy, but our findings suggest that antenatal care is not being provided according to the national guidelines.

The results of our study were consistent with previous studies in which higher age was significantly associated with diabetes or hypertension diagnoses [23,34,35]. Previous research on the associations between hypertension or diabetes and educational attainment has yielded mixed findings [23,30,34,37,42]. Our findings of no associations between educational attainment and hypertension or diabetes diagnoses contribute to the body of research attempting to better understand the nature of these relationships. Higher wealth has been consistently associated with hypertension and diabetes in previous studies [23,34,35]. Our findings support these previous estimates for hypertension, but not for diabetes. The most plausible explanation for this inconsistency is that this study focuses on a sub-population for whom the nature of the disease is different. While we asked the women in our study population about the timing of their diagnosis, we did not ask if their previous diagnosis was during a prior pregnancy. If the primary source of diabetes and hypertension screening for these women is antenatal care, then many of the diagnosed cases reported in this study may be transient gestational disease as opposed to chronic underlying conditions.

There are several limitations to this study. First, the absence of direct measurement or medical records to confirm self-reported information, coupled with low and unrepresentative screening coverage for diabetes, resulted in a high likelihood of misclassification of the women by disease status. Comparing the national data to our findings, up to half of the women with diabetes could have been misclassified in our study [22]. However, this study complements other studies that have estimated point prevalence with additional understanding about access to screening services and elements of antenatal care for women with known disease in rural contexts [29,31,35]. Second, women were asked about the timing of any diagnosis of hypertension or diabetes, but no further questions were asked to differentiate between gestationally and non-gestationally induced disease diagnoses. Self-reported diabetes diagnoses among respondents could have been due to gestational diabetes, type 1 diabetes, or type 2 diabetes. Self-reported hypertension diagnoses could have been associated with pregnancy-related hypertensive disorders like preeclampsia and gestational hypertension. Additional questions, if included in the survey, would have been subject to the same misclassification biases described above. From our experience and findings with this study, we conclude that differentiating between gestational and non-gestational diseases in this context would require a prospective study design. Third, the low rates of reported diabetes diagnoses made it difficult to detect differences in risk through our study and introduced the potential that differences observed could be due to Type II error or chance alone. For example, diabetes disease prevalence ranged from 8% among women with no education to 2% among women with post-secondary education, suggesting a possible higher risk among women with no education. This risk difference could be explained by differential use of the health system for preventative care, statistical error, or a true difference in disease risk. The goal of this study was not to precisely measure increased risk, but rather to explore and understand general patterns for further study. Further research to explore this difference in absolute prevalence in a larger population may be merited.

## Conclusion

Health care system constraints are a global challenge in addressing the burden of diabetes and hypertension. This study contributes to the global evidence base on the burden of diabetes and hypertension among childbearing women in low-income countries. Antenatal care provides an important opportunity for hypertension and diabetes screening among childbearing women. Focused efforts to ensure that women receive the recommended number of antenatal care contacts, coupled with improved compliance with antenatal care guidelines (including universal screening for diabetes at 24–28 weeks of pregnancy), would improve awareness of these diseases among women in their childbearing years in Bangladesh.

## Supporting information

S1 Checklist Inclusivity in global research checklist. (DOCX)

S1 Table Educational status of study participants, stratified by age in years, Baliakandi, Bangladesh, 2019. (DOCX)
