# Supplementary material for: Self-reported diabetes or hypertension diagnoses and antenatal care among child-bearing women in rural Bangladesh: A cross-sectional study
Source: PLOS Glob Public Health. 2023 Sep 14;3(9):e0002175. doi: 10.1371/journal.pgph.0002175 (PMC10501644; doi:10.1371/journal.pgph.0002175)
Supplement: S1 Table — (DOCX) [file pgph.0002175.s002.docx]

Table S1. Educational status of study participants, stratified by age in years, Baliakandi, Bangladesh, 2019.

|  | **Age (Years)** | | | |
| --- | --- | --- | --- | --- |
|  | **All women**  n(%) | **<20 years**  n(%) | **30-39 years**  n(%) | **40+ years**  n(%) |
| **Education (Completed)** |  |  |  |  |
| None | 202(4) | 10(1) | 102(10) | 13(33) |
| Primary | 873(19) | 94(10) | 316(31) | 15(39) |
| Secondary | 2,406(51) | 641(69) | 457(45) | 6(15) |
| Post-Secondary | 1,211(26) | 180(20) | 146(14) | 5(13) |
